# Israeli Medical Experts’ Knowledge, Attitudes, and Preferences in Allocating Donor Organs for Transplantation

**DOI:** 10.3390/ijerph19116945

**Published:** 2022-06-06

**Authors:** Amir Elalouf

**Affiliations:** Department of Management, Bar-Ilan University, Ramat Gan 5290002, Israel; amir.elalouf@biu.ac.il

**Keywords:** medical experts’ opinion, organ allocation, experts’ preference, organ allocating factors, hypothetical scenarios

## Abstract

Medical advancement has increased the confidence in successful organ transplants in end-stage patients. As the waitlist of organ demand is multiplying, the organ allocation process is becoming more crucial. In this situation, a transparent and efficient organ allocation policy is required. This study evaluates the preferences of medical experts to substantial factors for allocating organs in different hypothetical scenarios. Twenty-five medical professionals with a significant role in organ allocation were interviewed individually. The interview questionnaire comprised demographic information, organ donation status, important organ allocation factors, public preference knowledge, and experts’ preferences in different hypothetical scenarios. Most medical experts rated the waiting time and prognosis as the most important, while the next of kin donor status and care and contribution to the well-being of others were the least important factors for organ allocation. In expert opinion, medical experts significantly considered public preferences for organ allocation in making their decisions. Altogether, experts prioritized waiting time over successful transplant, age, and donor status in the hypothetical scenarios. In parallel, less chance of finding another organ, donor status, and successful transplant were prioritized over age. Medical experts are the key stakeholders; therefore, their opinions are substantial in formulating an organ allocation policy.

## 1. Introduction

Advancements in medical knowledge have developed the trust of end-stage organ failure patients in organ transplantation. The success rate of organ transplantation has significantly intensified the confidence that also increased the demand for vital organs [1]. According to the U.S. Organ Procurement and Transplantation Network (OPTN), 39,000 organs were transplanted in 2020. Still, 106,708 are waiting for an organ [2,3]. Organ shortage kills three Americans every day, and up to one in six of those waiting for a heart, liver, or lung transplant die or are too ill to be given organs. As the number of patients adds day-by-day on the waiting list for organ transplantation, the organ allocation procedure becomes more challenging. Different organ allocation policies are present but finding the most appropriate recipient is challenging. For kidney allocation, various policies have tried to balance utility (kidneys should be used as efficiently as possible) and equity (waitlisted patients have an equal chance of receiving kidneys) [4]. Nonetheless, substantial debates have raised many questions to balance the utility and equity, such as “Should we discriminate between patients while considering their medical conditions?” [5], but no one reported a definite settlement [6].

Transplantation represents a unique challenge for clinicians as they tend to care for many patients who could benefit from a similar donated organ. Donated organs must be deliberated as a national resource, and all the listed patients have an equal opportunity to receive the donated organ. Therefore, donated organ allocation and rights of competing recipients need to be clearly defined, focused, and evidence-based to merely benefit the patients [7]. Currently, organs from deceased donors are allocated based on criteria such as the likelihood of success, medical urgency, time on the waiting list, or pediatric status [8]. In the case of heart and liver transplants, clinicians’ decisions and centers’ policies prefer to allocate the organ to the most appropriate recipient. Some patients are listed as super-urgent; if they do not obtain the urgent transplant, it may lead to death so that patients receive the available donated organ with clear justification. Further, cold ischaemic time (CIT)—the interval between the cooling and implantation of an organ- is also considered in the organ allocation process. Organ allocated to patients with short CIT nearly available or present in a similar center [7].

Various organizations are managing organ transplantation in different countries, for instance, Euro-transplant in Europe, United Network for Organ Sharing (UNOS) in the U.S. [9], and Israel National Transplant Center (INTC) in Israel [10]. In leading countries, a point scoring system is practiced to allocate the kidneys. The patient who secures maximum scores, the available organ would be allocated to him/her. In this context, researchers have tried to improve the organ allocation system to make it transparent and effective. In 1990, David and Yechiali optimized the organ allocation model with different criteria to allocate various organs to recipients [11]. In 2001, Yuan et al. introduced a fuzzy logic-based kidney allocation system to deal with complexity and ambiguity near expert opinion [12]. Gundogar et al. (2005) established a kidney allocation system known as fuzzy organ allocation system (FORAS) and claimed it was better than other allocation systems [13]. Later, in 2008, a utility-based system was developed by Baskin and Nyberg to balance the demand and supply of kidney transplantation [14].

For liver allocation, a rule-based decision-making system was proposed by Cruz-Ramirez in 2013 [15]. In parallel, linear regression of score weights [16], fuzzy lung allocation system (FLAS) [17], Data Envelopment Analysis (DEA) [18], Delphi method, Analytic Hierarchy Process (AHP) [19,20,21], and Mamdani Style Fuzzy Inference System (MSFIS) [22] were developed and practiced for allocating organ in different regions at different time. Altogether, AHP and Delphi have been extensively used for developing the organ allocation system [19,20,21]. Recently, Taherkhani et al. used the Intuitionistic Fuzzy AHP method for weighting kidney allocation criteria to remove the uncertainty in decision making [9]. Related studies pointed out that sometimes, more deserving candidates for allocation are inappropriate, such as those with alcoholism, HIV, suicidal tendencies, or drug addiction [23]. Therefore, medical factors such as inoperable coronary artery disease, active systemic lupus erythematosus, and diabetes are also considered as transplantation contraindications [24].

Allocating organs is a societal undertaking, as preferring a particular patient always implies deselecting others who may not be eligible for future organ transplants. The priority-setting and decision-making depend on various stakeholders, including the general public (who are the potential organ suppliers who influence the availability of organs for transplantation), medical professionals (who are responsible for patient care), and the patients (who will receive the new organs) [25]. Medical health experts play a crucial role in the organ allocation process. They communicate with the patients, explain the procedure, implement the administrative proceedings, evaluate the transplant need, and perform the mandatory medical actions for transplantation [24].

Johri and Ubel [26] claimed that transplantation policy should not blindly reflect the perception and specific standards of transplantation professionals and healthcare researchers, who generally come from different backgrounds than most general public members. Nor should such policies solely reflect the attitudes and morals of the general public.

### 1.1. Public Preferences regarding Organ Allocation

Transplantation relies on public needs and people’s willingness to donate; hence it is vital to involve the general public preferences in organ allocation decisions [27]. The general public broadly agrees that organs should be preferentially allocated to candidates expected to benefit from them the most regarding life expectancy and quality of life. In numerous studies, maximum benefit caused the slightest moral discomfort among survey respondents and was assessed as the main parameter in selecting transplant recipients. Typically, survey respondents view time spent on the waiting list as an essential criterion in organ allocation. They believed that priority should be given to candidates who have been waiting a long time for a transplant. Noticeably, this factor is more objective and unequivocal than other criteria; hence people feel less ambivalent when integrating it into their decisions [28,29,30,31,32]. Furthermore, time spent on the waiting list is perceived as culturally acceptable and treated as an ‘automatic’ parameter invoking a systematic and mechanical procedure [32].

Regarding age, members of the general public have indicated that, when organ availability is limited, the young should be prioritized over the elderly. The public deemed that younger people should have an opportunity to live and anticipated that they would have a better prognosis than older individuals [28,30,31,33,34]. Nevertheless, a survey conducted in Israel [29] discovered that respondents did not perceive recipient age as a criterion that should be attributed excessive importance. In fact, 24% of the interviewees ranked it as the least essential factor in determining transplantation priorities.

The notion of registered donors’ preferred status gained community members’ support as respondents felt that registered donors’ should be prioritized [31,33]. Nonetheless, Israeli respondents ranked donor status as the least important criterion in allocation decisions despite registered donors’ prioritization by Israel’s organ allocation policies [29]. Moreover, according to survey results, members of the general public feel aversion toward any preferences based on variables such as recipient gender, ethnicity, social utility, employment status, occupation, ability to pay, and socioeconomic status. They believe that these variables should not impact a patient’s access to the waiting list and impede chances of receiving transplantation [31,35,36].

Studies highlighted that some traits impact likelihood and priority ratings due to different social characteristics’ effects (e.g., gender and ethnicity) on values and preferences. For instance, Sears et al. (2000) learned that race has a particular impact on decision-making. They found out that compared with Caucasians, African Americans believe that every person who needs transplantation should be given high priority [36]. Similarly, Clark et al. (2009) noted that preferences slightly varied according to gender; notably differed according to ethnic origin. Caucasians, non-white, and South Asian ethnic minorities were not inclined to give precedence to recipients with a good tissue match and tended not to prioritize younger recipients. Further, non-white and South Asian ethnic minorities were less likely to prioritize people with moderate rather than severe diseases that affect life expectancy [37].

To conclude, studies have proved that the general public holds opinions regarding the prioritization of transplant candidates. These preferences are based on a delicate balancing act of expediency, morality, socioeconomic aspects, justice, and equitable ideals [31]. Decisions and comparative significance of different allocation factors influence equality; therefore, they are constantly debated. Thus, it is interesting to observe the opinions of the crucial players in charge of the transplant process.

### 1.2. Health Care Professionals’ Preferences regarding Organ Allocation

Transplantation and organ donation require organization, coordination, planning, managing registries, waitlists, and effective resource allocation. Health care professionals identify potential donors, seek families’ consent, conduct suitability tests, and coordinate with transplant authorities to find a match. Therefore, medical professionals hold the key to improving the organ transplantation process as they stand on the front line in health care [38].

Previous studies assessed the medical expert’s opinion on organ allocation by agreeing/disagreeing with the statements [39,40] and testing the eligibility of hypothetical patients [41]. Usually, comparing and ranking criteria for allocation eligibility regarding the importance of patients is rarely executed. However, the interviews are often exploratory [42,43]. In this regard, Thamer et al. determined the U.S. nephrologists’ recommendations regarding eight unique hypothetical patient scenarios. Patients with end-stage renal disease who were female, Asian, and not black were more likely to be recommended for renal transplantation. According to some other researchers, the well-documented disparities in kidney transplantation between black and white people may stem from unaccounted-for factors or result from subsequent steps in the transplantation process [41]. Cass et al. elucidated what factors influence medical professionals’ decisions about patients’ suitability for kidney transplants. Experts recommended young and regular weight recipients over smokers, heart disease, and diabetes patients [39].

Tong et al. interviewed the nephrologists of 15 Australian transplant and nephrology centers. They emphasized that the primary responsibility is to give their patients easy access to transplantation (e.g., waitlist of patients and acceptance of individual kidneys), maintain transparency, avoid value judgments, and uphold professional integrity. Furthermore, they stipulated that the allocation system should comprise age compatibility so that younger candidates had a higher chance of receiving a more youthful and better-quality kidney. In addition, nephrologists advocated maximizing transplant survival since it was perceived as the primary community benefit. An interesting finding of the study was that although nephrologists had personal views about societal benefit and equity, they believed that the issue of resolving this equilibrium should be an external responsibility borne by policymakers and the general public [42].

Davison, Kromm, and Currie (2010) [44] found that health care professionals favored a more utilitarian to egalitarian approach in allocating deceased donor kidneys. They preferred match ability to equity (first come, first served), believing that a functional approach is needed to allow maximum flexibility in accommodating advances in renal transplants. On the topic of precedence in organ donation, research which was executed in India by Almeida et al. (2016) [40] discovered that a substantial majority of medical professionals (90.7%), who took part in the survey, upheld giving priority to organ donors in the event of a future need for an organ. Specifically, 47.1% of the participants stated that donors should be rewarded and recognized for their selfless and humane acts.

Differences in priorities among stakeholders, namely, considerable variance in the preferences of members of the general public, family physicians, and hospital clinicians, were presented in a survey by Neuberger et al. [45]. While respondents representing the general public prioritized age, transplant outcome, and time on the waiting list, family physicians maintained that transplant outcome, age, and likely work status after transplantation were the most important criteria. Hospital clinicians evaluated transplant outcomes, work status, and non-involvement of substance abuse as the most critical aspects. Nonetheless, all three groups agreed that anti-social behavior and substance abuse should hinder entitlement to transplantation.

Regarding willingness to donate organs, Bedenko et al. [46] assessed the knowledge and acceptance of the general public and professionals working in intensive care units. Their research results revealed the tendency to donate organs was substantially higher among the health professionals group. Further, in comparison with males, females were more willing to donate. Yet, no significant difference was found concerning religion, education level, or income. 

In a survey performed in Germany among intensive care specialists [47], 81% of respondents favor organ donation in the event of brain death. The consent rate in the medical profession was 84% compared to 75% in the nursing profession. However, only 45.3% of the participants (47% of physicians and 44% of nurses) have signed an organ donor card in practice. Merely 45% shared and confided their decision and preference for organ donation with family and friends.

Notwithstanding, some nephrologists believed that they had a duty to protect their centers’ reputations by selecting “good” patients, which caused frustration. The nephrologists preferred to maximize societal benefit while ensuring equity but did not want direct responsibility for this across the entire health care system. In contrast, they were responsible for resolving potential tensions between policymakers and the community [42]. In a related study, health professionals’ attitude was examined regarding the controversial issues of transplantation. Most experts disagreed with giving incentives to donors and allocating organs to HIV/hepatitis carriers. For this purpose, the majority favored allocating organs to donors due to their previous acts [40].

In summary, given that the views of medical professionals largely shape allocation policies, it is of interest to recognize whether their preferences diverge from those of the general public and if different groups of medical professionals hold different opinions.

The present study is the continuation of a previous study [29], where the Israeli public’s preferences for organ allocation were correlated with the current organ allocation policies. Medical professionals are crucial stakeholders in the organ allocation process. They are responsible for one’s life. Therefore, medical experts’ opinion is significant in the organ allocation process. To the best of our knowledge, no other study evaluated the Israeli medical experts’ preferences while considering the crucial factors in organ allocation policies. Hence, this study (1) evaluates the medical experts’ preferences for different factors in the organ allocation process; (2) analyzes the experts’ opinions about the most and least important factors in organ allocation; (3) investigates the importance of other stakeholders, for instance, public preferences compared to medical experts; (4) assesses professionals’ knowledge about the most, second, and least essential factor considered by the public in organ allocation and; (5) compares experts’ preferences about the hypothetical cases for organ allocation to the public preferences and the Israeli National Transplant Center’s point system.

## 2. Materials and Methods

### 2.1. Study Design

Individual interviews were conducted with decision-making healthcare professionals in organ allocation with the consent of the Organ Allocation Association. The Research Ethics Committee of the university department approved the study (Ref No. NIHP 2016/78).

### 2.2. Participants and Eligible Criteria

The inclusion criteria for this study were practitioners linked to organ allocation decision-making and the transplantation process (e.g., nephrologists, surgeons, coordinators, nurses, ethics specialists). Respondents were chosen according to their expertise, knowledge, and comprehension of transplant criteria. Subsequently, this study ranked the participants’ replies by creating a descriptive and inferential analysis of respondents’ attributes and preferences regarding donor organ distribution. Finally, the paper investigated any inconsistencies between reported choices and theoretical allocation decisions. We contacted twenty-five medical experts with at least 15 years of professional experience. In addition, the interviewees hold a position on the organ allocation and transplant decision-making committee for more than five years, for instance, as chairman of the association of kidney patients and their families, nephrologists, surgeons, physicians, nutritionists, nephrology nurses, transplant coordinators, ethics and law professors, and human rights coordinators. The interviewees were further divided into two groups according to their role in the organ allocation and transplant decision-making committee (i.e., transplant health care professionals and organ allocation and transplant managers).

### 2.3. Interview and Questionnaire

All the participants involved in the decision-making process about organ allocation were interviewed individually through a telephone and online survey to attain the research aims. A questionnaire was administered to a representative sample of medical professionals and experts practicing in Israel. The questions were validated, and pilot tested on nephrologists. An investigator conducted a telephonic qualitative interview with each participant in their free time. The questionnaire was designated into four sections with straightforward questions. Section 1 comprises personal information such as gender, age, area of expertise, marital status, number of children, health status, place of birth, religion, religiosity, and financial status of each interviewee. In Section 2, organ allocation factors such as recipient’s age, care and contribution to the well-being of others, waiting time, prognosis, the chance of receiving another organ, and signing an organ donor card were rated from 1 to 7 (1 means not at all important and seven means very important). The mean interval of 1–1.86 is considered as not at all important, 1.86–2.71 as moderately not important, 2.71–3.57 as slightly not important, 3.57–4.43 as neutral, 4.43–5.29 as slightly important, 5.29–6.14 as moderately important, and 6.14–7.00 as very important. In Section 3, medical experts were asked for their most and least important factors for organ allocation. Then, they were asked about the importance of public preferences in their decision. Their knowledge about the most, second, and least important factors considered by the public in the organ allocation process was also tested. In the last section considering all the preferences, eleven hypothetical organ allocation scenarios were presented to evaluate the right person for transplantation. In each case, two candidates with diverse characteristics were portrayed. The participant had to select which of the two should receive an organ or select “no preference”.

### 2.4. Statistical Analysis

All the responses were arranged in Microsoft Office Excel Spreadsheet. SPSS version 21 was used for applying descriptive and inferential statistics. Frequencies, percentages mean, median, and standard deviation were calculated in descriptive statistics. The chi-squared test compared and the Pearson test correlated the categorical variables in inferential statistics while considering the confidence level of 95%.

## 3. Results

### 3.1. Demographic Distribution and Organ Donation Status

The demographic information of the participants in the study was analyzed in descriptive statistics by frequencies, percentages, mean and standard deviation. Table 1 shows the demographic characteristics of the medical experts involved in the study. Twenty-five medical professionals decided on the organ allocation to the most deserving recipient. The males and females were present in a 1:1.083 ratio. The average age range was 35–83 years (M = 50.52, SD = 12.04). All the participants in the study have medical education with a specialization in their fields. They have held a position on the organ allocation and transplant decision-making committee for more than five years. Most of the participants were nephrologists. The study classifies nephrologists, surgeons, and physicians as transplant health care professionals. The chairman of the association of kidney patients and their families, human resource coordinator, nutritionist, nephrology nurse, transplant coordinator, and an ethical professor are categorized as management and organization due to the managerial nature of their position in the organ allocation and transplantation procedure. Lastly, registered organ donors’ frequency was determined and compared with other demographic parameters using the chi-square test, shown in Table 2.

### 3.2. Declared Preferences for Organ Allocation Criteria

**Figure 1:**
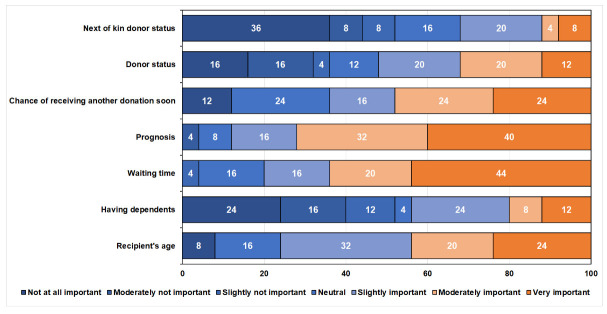
Importance of organ allocation parameters in percentage.

In the second part of the interview, experts’ attitudes and preferences were examined by rating the most important factors for organ allocation (Table 3, Figure 1). The prognosis criterion was rated as a moderately important parameter for organ allocation. A chi-square test of independence also revealed that prognosis was considered a highly significant parameter over others, χ^2^ (4) = 12, *p* = 0.01. In contrast, the chi-square test of independence showed a significant association between the interviewee’s role in organ allocation and the recipient’s age, χ^2^ (4) = 10.3, *p* = 0.03. Similarly, waiting time was also considered an important and significant parameter, χ^2^ (4) = 10.8, *p* = 0.03. The mean of the experts’ opinion was slightly unimportant (M = 3.2, SD = 2.06) for the patient’s next of kin donor status but significantly different from the others, χ^2^ (6) = 12.8, *p* = 0.04. Pearson rank-order correlation was used to examine the relationship between the parameters for the organ allocation. There was a positive significant correlation between having dependents (i.e., family responsibilities), and the recipient’s medical history, i.e., a previous disease (r_s_ = 0.40, *p* = 0.04), waiting time, and the chance of receiving another donation shortly (r_s_ = 0.49, *p* = 0.01), and the recipient’s medical history and the patient’s next of kin donor status (r_s_ = 0.61, *p* = 0.001).

The study also examined experts’ opinions about the most and least important organ allocation criteria. Table 4 represents the expert’s opinion about the most and least important factors for organ allocation. The results implied that professionals perceived prognosis as the most important parameter and donor status as the least important criterion. Specifically, 52% of the professionals gave precedence to prognosis, and the second most widespread choice was waiting time (24%). In contrast, 60% specified donor status as the least essential criterion. It is noteworthy that the donor status criterion yielded similar results among registered and non-registered donors.

A chi-square test of independence confirmed the positive significance among the most essential factors, χ^2^ (3) = 10.68, *p* = 0.01, and a high level of significance among the least important factors, χ^2^ (3) = 17.72, *p* = 0.001. According to experts’ views, there was no significant correlation between the most and least important factors. Table 4 presents the findings.

### 3.3. Experts’ Hypothesis about the General Public Preferences

**Figure 2:**
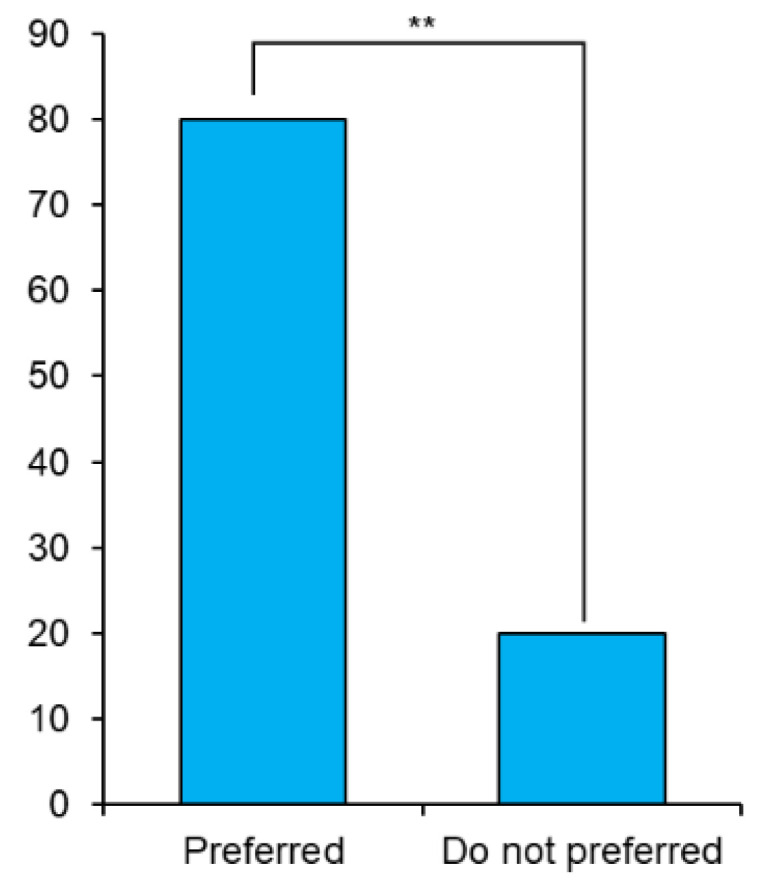
Experts’ percentage that considered public preferences in the organ allocation process (** *p* < 0.01).

Figure 2 represents the highly significant percentage of experts who evaluated and considered the general public procedure in the organ allocation process, χ^2^ = 9, *p* = 0.003. Table 5 shows experts’ estimations of the general public perspectives. Most experts selected waiting time as most important, age as second important, and donor status as the least important factor in the general public opinion. A high level of significance among the most important (χ^2^ (5) = 25.16, *p* < 0.01) and least important (χ^2^ (4) = 14.4, *p* = 0.006) factors was confirmed by chi-square test of independence. Meanwhile, Pearson rank-order correlation established positive significant relationship between most important and second important factor (r_s_ = 0.43, *p* = 0.02), most important and least important factors (r_s_ = 0.46, *p* = 0.01), and second important and least important factors (r_s_ = 0.49, *p* = 0.01).

### 3.4. Comparison among Experts’ Preferences, Public Preferences, and Declared Preferences

A previous study [29] inquired about the correlation between the Israeli public’s preferences for organ allocation and the present organ allocation policies. Here, medical field experts’ preferences for organ allocation have been compared with the Israeli public’s priorities and current allocation policies (presented in Table 6). The experts’ preferences were consistent with most public preferences but diversified from allocation policies. Experts mainly emphasized the prognosis and waiting time and did not entrust importance to the donor’s status and age. For instance, experts (84%) chose no preference when asked about a 30-years-old candidate or a 45-years-old candidate. Although, an equal number of experts preferred both candidates (χ^2^ = 28.88, *p* < 0.001).

Similarly, experts showed preference to waiting time over the chance of a successful transplant in scenario 2 (60%, χ^2^ = 24, *p* < 0.001), and age in scenario 3 (80%, χ^2^ = 24.56, *p* < 0.001). In contrast, regarding lesser odds of finding another suitable organ soon, especially kidneys, 52% of experts selected the probability criterion, and 44% preferred waiting time as in scenario 11 (χ^2^ = 9.92, *p* < 0.01). Experts prioritized the patients who had signed organ donor cards (44%) over the age parameter, as shown in scenario 5. However, donors were not given priority over a lesser chance of getting another organ (χ^2^ = 21.44, *p* < 0.001, in scenario 6), waiting time (t (24) = 31.659, *p* < 0.01, in scenario 8), and the chance of a successful transplant (χ^2^ = 2.96, in scenario 10). Further, the significant preference for a successful transplant over the odds of finding another suitable organ soon (χ^2^ = 33.68, *p* < 0.001, in scenario 4) and age (χ^2^ = 5.84, *p* < 0.05, in scenario 7) were observed. While highly significant preference (76%) for lesser odds of finding another suitable organ soon over advanced age (χ^2^ = 20.48, *p* < 0.001) was found in scenario 9.

Table 6 also represents the divergence among the experts’ preferences, public preferences, and declared preferences. For instance, in scenario 2, experts (60%) preferred waiting time over a successful transplant. In comparison, the public and the point system preferred the chance of a successful transplant. Similarly, in scenario 5, most experts preferred donor status over the age criterion. Nevertheless, 40% of the professionals and 41% of the public did not show preference, unlike the declared preference. Both patients had identical point scores, according to the national point system. Further, experts preferred the chance of finding another donor in scenarios 6, 9, and 11. In contrast, 40% of the public preferred registered donors, 36% preferred the same as the experts in scenario 6 (where the point system scored the same points for both patients), and the point system preferred younger patients in scenario 9 and waiting time in scenario 11.

### 3.5. Willingness to Be an Organ Donor

As mentioned in Table 2, only three male participants were not organ donors. The three interviewees were asked about their willingness to sign an organ donor card shortly. One said he would “definitely” sign a donor card soon. Another replied “probably not” in response to signing an organ donation card, even if his preferences will be ranked as highly important in organ allocation policies. The last one stated he would probably sign the organ donation card shortly and “definitely” if his inclinations are ranked as highly important in organ allocation policies.

## 4. Discussion

Medical health professionals are more reluctant to decide about organ allocation based on the equity and efficiency of a successful transplant. The main aim of the policy is transparency in organ allocation that enhances the benefits to the community [42]. For instance, the U.S. Organ Procurement and Transplantation Network formulated a policy prioritizing waiting time in kidney allocation [49]. Based on previous data [50], age was given preference by saying “old-for-old” in order to allocate younger kidneys to the younger and older kidneys to elderly people [51,52]. However, in 2011, the U.S. changed the organ allocation preference toward predicted successful and long-lasting kidney graft survival [53]. 

This study contributed to the organ allocation policy in Israel by adding medical experts’ preferences to different significant factors that should be considered in organ allocation. Based on previous studies [39,41,49,50,51,52,53,54,55], the paper prioritized factors such as prognosis, recipient’s age, care and contribution to the well-being of others, waiting time, the chance of receiving another kidney, and donor status. These parameters are significant in organ allocation policies. Most experts significantly rated waiting time and prognosis as essential factors (Figure 1). Comparably, in a different section of the questionnaire, the experts selected these factors as the most crucial (Table 4). The hypothetical cases in scenarios 2, 3, and 8 also reflect the experts’ preference for waiting time, i.e., the waiting time criterion was prioritized over a successful transplant, age, and donor status (Table 6). Experts’ preferences in a 2005 U.S. Organ Procurement and Transplantation Network policy were the same as this study’s findings [49]. Similarly, in another study, the public rated waiting time as the most prioritized factor [56].

The experts rated age as a moderately important factor (Figure 1 and Table 3). However, in another section, 20% of the experts selected this parameter as the least important factor for organ allocation. The results of selecting the recipient for the organ were also reflected in scenarios 1, 5, 7, and 9. Age was not prioritized over donor status, successful transplant, and finding another suitable organ soon. It is noteworthy that age was not a significant factor in U.S. policy before 2005 [51,52].

Further, a study reported less priority to age over waiting time [56]. In comparison, research reported a recommendation of nephrologists to allocate organs to the youngers [39]. In another study, compliant patients were six times more recommended by the nephrologists [41]. The chance of a successful transplant was also prioritized over finding another suitable organ shortly, age, and non-registered donors in scenarios 4, 7, and 10, respectively. This supports the Australian organ allocation criteria reported in a study [39].

Organ donors significantly reflect the decision-making to allocate the organ to the right person. The person donating the organ would like his/her organ to be allocated after following transparent procedures. Accordingly, this study evaluated the organ donor status of all the candidates who participated in the study. Organ donor status was compared with the demographic information (Table 2). The chi-square comparison represented that birthplace and financial status had significantly affected the participants’ donor status. Namely, an above-average financial status and origin (i.e., native Israelis) impact the willingness to be an organ donor.

The three non-donors in the study were nephrologists. One recipient will sign the donor card soon, while another is disinclined to sign. Nevertheless, the third expert would probably sign the card soon and “definitely” if the transplantation policy incorporates his preferences. This study recognized substantial seriousness among medical experts in organ donation. Thus, including the experts’ opinions in formulating the organ allocation policy might have positive consequences.

As medical experts, the public is a stakeholder in formulating an organ allocation policy. Many studies had already given significant weightage to the general public preferences [25,29,45,56] and different communities such as scholars [34], patients [25], adult and pediatric donors [57], academic and non-academic employees of educational institutions [30], family doctors, and gastroenterologists [45]. Therefore, this study evaluated the importance of public preferences to experts while allocating organs and then tested their knowledge about the public inclinations. This study found that experts significantly correlated with public opinion (Figure 2). The experts predicted the general public preferences (Table 5). A previous study that evaluated the community tendencies [29] reported prognosis and waiting time as the most significant parameters and donor status as the least essential factor for organ allocation.

## 5. Limitations and Future-Prospects

This study is limited to the five significant factors in organ allocation policy. However, the study added the Israeli medical health professionals’ preferences in organ allocation procedures. Further, the study has several limitations.

The scenarios were inadequate and generated conflicts between allocation decisions and declared policy. Other factors such as recipient lifestyle must be considered for a clear and feasible allocation decision. For instance, a study reported the concerns of medical professionals related to the illegal activities of the recipient [40]. Similarly, a systematic review stated the principles of community preferences to organ allocation factors such as life quality, social valuation, moral deservingness, prejudice, first come, first served, and medical emergency [31]. In another study, treatment adherence, life gains quality, and social productivity were substantially preferred for transparent and efficient allocating policy [23]. Further, patient advocacy, professional integrity, center reputation, social benefits [27,58], enforcement, relevance, appeal [43], medical background, sociodemographic status [59], and religious conviction [60] are the cluster of factors substantial for making an improved allocation system that attracts significant organ donors.

According to the Israel Ministry of Foreign Affairs, approximately 32,000 medical doctors and 54,000 registered nurses are working [61]. However, this study evaluated minimal medical health professionals’ opinions on organ allocation policy. In contrast, this study’s medical professionals have significant roles and experience in making-decision to the organ allocation process.

The present study is based on the Jewish population. In contrast, according to the Jewish Virtual Library Latest Population Statistics for Israel [62], other populations like Muslims (18%), Christian (2%), and Druze (2%) are an intrinsic part of the country, and their concerns are also crucial in making organ allocation policies. Thus, we ought to consider their preferences in future studies.

In this study, different scenarios were composed without noticing the point system decision and public preferences. That is why some data were missing in Table 6. We shall revise these scenarios in future studies to amend the research gap.

Lastly, this study did not evaluate the wide range of hypothetical scenarios such as age, waiting time, successful transplant, and finding another organ shortly. A wide range of these factors must be evaluated for allocating organs in future studies.

## 6. Conclusions

Formulating an organ allocation policy is a crucial process in which all the factors and stakeholders’ preferences have a substantial role. Medical professionals linked with organ transplantation are significant donors and are ready to sign the donor card. Meanwhile, medical experts considered the general public’s opinions and preferences regarding crucial factors. Prognosis and waiting time were found to be the most significant factors in organ allocation. In contrast, age and donor status were not decisive factors in allocating organs. In closing, other essential factors and their wide range in different scenarios must be considered in formulating a transparent and efficient organ allocation policy.

## Figures and Tables

**Table 1:** The demographic characteristics.

Demographic Characteristics		Number	Percentage
Gender	Male	12	48
	Female	13	52
Profession	Nephrologists	14	56
	Surgeons	3	12
	Physicians	2	8
	Others	6	24
Role in organ allocation	Health care professionals	19	76
	Organ allocation managers	6	24
Religion	Secular	18	72
	Traditional	4	16
	Orthodox	1	4
	Religious	2	8
Financial status	Average	3	12
	Above-average	16	64
	Slightly above average	6	24

**Table 2:** Comparison of demographic data with registered organ donors.

Demographic Characteristics		Yes N (%)	No N (%)	Chi-Square Value	*p*-Value
Gender					
	Male	11 (44)	1 (4)	0.294	0.58
	Female	11 (44)	2 (8)		
Age (y)					
	35–50	12 (48)	2 (8)	349	0.84
	51–65	8 (32)	1 (4)		
	>65	2 (8)	0 (0)		
Profession					
	Surgeons	3 (12)	0 (0)	2.679	0.95
	Nephrologists	11 (44)	3 (12)		
	Human resource coordinator	1 (4)	0 (0)		
	Chairman of association of kidney patients and their families	1 (4)	0 (0)		
	Physicians	2 (8)	0 (0)		
	Nutritionist	1 (4)	0 (0)		
	Nephrology nurse	1 (4)	0 (0)		
	Transplant coordinators	1 (4)	0 (0)		
	Ethical professor	1 (4)	0 (0)		
Role in organ allocation	Health care professionals	16 (64)	3 (12)	1.077	0.299
	Organ allocation managers	6 (24)	0 (0)		
Health status					
	Excellent	8 (32)	1 (4)	2.694	0.61
	Very good	6 (24)	0 (0)		
	Good	4 (16)	1 (4)		
	Fair	2 (8)	1 (4)		
	Poor	2 (8)	0 (0)		
Marital status					
	Married	19 (76)	3 (12)	0.465	0.49
	Unmarried	2 (8)	0 (0)		
Children					
	0	7 (28)	1 (4)	7.244	0.203
	1	1 (4)	1 (4)		
	2	4 (16)	0 (0)		
	3	8 (32)	0 (0)		
	4	1 (4)	1 (4)		
	5	1 (4)	0 (0)		
Birthplace					
	Israel	16 (64)	1 (4)	9.774	0.008
	America	5 (20)	0 (0)		
	USSR	1 (4)	2 (8)		
Immigrant					
	Parents	8 (32)	0 (0)	2.286	0.131
	Self	6 (24)	2 (8)		
Religion					
	Jewish	21 (84)	3 (12)	0.142	0.706
	Christian	1 (4)	0 (0)		
Religiosity					
	Secular	16 (64)	2 (8)	1.741	0.783
	Religious	2 (8)	0 (0)		
	Traditional	3 (12)	1 (4)		
	Orthodox	1 (4)	0 (0)		
Financial status					
	Above average	15 (60)	1 (4)	9.809	0.007
	Average	1 (4)	2 (8)		
	Slightly above average	6 (24)	0 (0)		

**Table 3:** Analysis of organ allocation criteria.

	Mean ± SD	Median	Chi-Square Test	*p*-Value	Pearson Correlation
1 Recipient’s age	5.2 ± 1.63	5	4	0.40	
2 Having dependents	3.6 ± 2.14	3	6.08	0.41	2 vs. 6 (*p* = 0.04)
3 Waiting time	5.84 ± 1.28	6	10.8	0.03	3 vs. 5 (*p* = 0.01)
4 Prognosis	5.92 ± 1.26	6	12	0.01	
5 Chance of receiving another donation soon	5 ± 1.87	5	1.6	0.8	
6 Donor status	4.12 ± 2.09	5	3.28	0.77	6 vs. 7 (*p* = 0.001)
7 Next of kin donor status	3.2 ± 2.06	3	12.8	0.04	

**Table 4:** Medical experts’ views about the most and least important factors in organ allocation.

	Chance of Receiving Another Donor N (%)	Waiting Time N (%)	Prognosis N (%)	Age N (%)	Donor Status N (%)	Chi-Square	*p*-Value
Most Important	3 (12)	6 (24)	13 (52)	3 (12)	0	10.68	0.01
Least Important	4 (16)	1 (4)	0	5 (20)	15 (60)	17.72	0.001

**Table 5:** Expert’s estimations about the general public perspectives.

	Chance of Receiving Another Organ N (%)	Waiting Time N (%)	Prognosis N (%)	Recipient’s Age N (%)	Donor Status N (%)	Don’t Know N (%)	Chi-Square	*p*-Value	Pearson Correlation
1 Most Important	1 (4)	13 (52)	2 (8)	3 (12)	1 (4)	5 (20)	25.16	0.00	1 vs. 2 (*p* = 0.02)
2 Runner-up	3 (12)	4 (16)	5 (20)	7 (28)	1 (4)	5 (20)	5	0.41	2 vs. 3 (*p* = 0.01)
3 Least Important	7 (28)	0 (0)	1 (4)	1 (4)	11 (44)	5 (20)	14.4	0.006	3 vs. 1 (*p* = 0.01)

**Table 6:** Comparison of medical experts’ preferences, reported public preferences, and Israeli National Transplant Center’s point system.

Scenario no.	Description	Experts’ Preferences	Public Preferences [29]	The Point System Decision [48]
1	Patient A is 30 years old. Patient B is 45 years old.			
	No Preference	84%	71.5%	Patient A 2.9 points
	Patient A	8%	20%	
	Patient B	8%	5%	Patient B 1.4 points
2	Patient A has a 70% chance of a successful transplant and waiting for 4 years. Patient B has a 90% chance of a successful transplant and waiting for 1 year.			
	No Preference	0	12%	Patient A 3.92 points
	Patient A	60%	39%	
	Patient B	40%	45%	Patient B 4.48 points
3	Patient A is 45 years old and waiting for 2 years. Patient B is 35 years old and waiting for the last 6 months.			
	No Preference	12%		Patient A 2.46 points
	Patient A	80%		
	Patient B	8%		Patient B 2.7 points
4	Patient A has a 90% chance of a successful transplant. The odds of finding another suitable kidney shortly (if he does not receive the kidney now) are 70%. Patient B has an 80% chance of a successful transplant. The odds of finding another suitable kidney soon (if he does not receive the kidney now) are 30%.			
	No Preference	4%		
	Patient A	8%		
	Patient B	88%		
5	Patient A is 40 years old and signed an organ donor card. Patient B is 20 years old and had not signed an organ donor			
	No Preference	40%	41%	Patient A 3.9 points
	Patient A	44%	36%	
	Patient B	16%	20%	Patient B 3.9 points
6	Patient A is a registered donor and has a 70% chance to obtain another suitable kidney shortly if he does not receive the kidney now. Patient B is not a registered donor and has a 30% chance to obtain another suitable kidney shortly if he does not receive the kidney now.			
	No Preference	4%	21%	Patient A 4 points
	Patient A	20%	40%	
	Patient B	76%	36%	Patient B 4 points
7	Patient A is 24 years old. He has a 70% chance of a successful transplant. Patient B is 50 years old. He has a 90% chance of a successful transplant.			
	No Preference	24%		Patient A 3.5 points
	Patient A	20%		
	Patient B	56%		Patient B 1.04 points
8	Patient A is an organ donor and waiting for 1 year. Patient B is not an organ donor and waiting for 4 years.			
	No Preference	0	21%	Patient A 2.48 points
	Patient A	24%	23%	
	Patient B	76%	53%	Patient B 1.92 points
9	Patient A is 40 years old. If he does not receive a kidney now, there is an 80% chance of finding another suitable kidney soon. Patient B is 55 years old. The odds of finding another suitable kidney shortly (if he does not receive the kidney now) are 40%.			
	No Preference	12%		Patient A 1.99 points
	Patient A	12%		
	Patient B	76%		Patient B 0.56 points
10	Patient A is a registered organ donor and has a 70% chance of a successful transplant. Patient B is not a registered organ donor and has a 90% chance of a successful transplant.			
	No Preference	20%	19%	Patient A 4 points
	Patient A	32%	27%	
	Patient B	48%	51%	Patient B 4 points
11	Patient A has been waiting for a month. The odds of finding another suitable kidney soon (if he does not receive the kidney now) are 30%. Patient B has been on the waiting list for two years. The chances of finding another suitable kidney shortly (if he does not receive the kidney now) are 60%.			
	No Preference	4%		Patient A 0.04 points
	Patient A	52%		
	Patient B	44%		Patient B 0.96 points

## Data Availability

The data presented in this study are available on request from the corresponding author. The data are not publicly available due to privacy issues.
